# Biological Deep Temperature Imaging with Fluorescence Lifetime of Rare-Earth-Doped Ceramics Particles in the Second NIR Biological Window

**DOI:** 10.1038/s41598-019-49291-x

**Published:** 2019-09-05

**Authors:** Takumi Chihara, Masakazu Umezawa, Keiji Miyata, Shota Sekiyama, Naoki Hosokawa, Kyohei Okubo, Masao Kamimura, Kohei Soga

**Affiliations:** 1 Department of Materials Science and Technology, Faculty of Industrial Science and Technology, Tokyo University of Science, 6-3-1 Niijuku, Katsushika, Tokyo, Japan; 2 Imaging Frontier Center (IFC), Research Institute for Science and Technology (RIST), Tokyo University of Science, 2641 Yamazaki, Noda, Japan

**Keywords:** Fluorescence imaging, Biophotonics

## Abstract

Contactless thermal imaging generally relies on mid-infrared cameras and fluorescence imaging with temperature-sensitive phosphors. Fluorescent thermometry in the near-infrared (NIR) region is an emerging technique for analysing deep biological tissues but still requires observation depth calibration. We present an NIR fluorescence time-gated imaging (TGI) thermometry technology based on fluorescence lifetime, an intrinsic fluorophore time constant unrelated to observation depth. Fluorophore used is NaYF_4_ co-doped with Nd^3+^ and Yb^3+^ that emits fluorescence at 1000 nm. An agarose gel-based phantom with the fluorophore embedded at a 5-mm depth was covered by sheets of meat to vary the observation depth. The temperature was determined independently from depth by sequences of NIR fluorescence decay images, and the rate of change in the fluorescence lifetime per temperature was almost constant (−0.0092 ~ −0.010 °C^−1^) at depths ranging from 0 to 1.4 mm of meat, providing non-contact and absolute measurements of temperature in deep biological tissues.

## Introduction

There is considerable interest in the development of temperature measurements for use in biomedicine since conventional contact thermometries, such as thermocouples and thermometers, are unsuitable for remote temperature measurement^1^. Because temperature is highly important for controlling various biological functions, including growth^2^, response^3^, and cell division^4^, the development of contactless thermometries is important to visualize dynamic thermal changes *in vivo* and is also necessary for thermal therapies^5–7^.

Recently, fluorescence imaging has been developed for contactless thermometry applications^8–11^ since temperature-dependent changes in the fluorescence lifetime of materials such as rare-earth-doped ceramics particles^12^, carbonous compounds^13^, and Cr^3+^-activated compounds^14^ have been reported. The fluorescence lifetime is calculated from the decay rate and is unperturbed by the absolute intensity. Thus, thermometry based on the change in fluorescence lifetime is free from fluctuations in excitation power or in concentration-dependent output^15–17^. Temperature imaging using lifetime thermometry has been reported previously^13,18^; however, use of ultraviolet (UV) and visible (VIS) light limits the measurable depth, and thermometers that detect mid-infrared are limited by the optical loss from biological tissues. Only surface temperature can be measured by the current methods.

For temperature imaging, fluorescence in the near-infrared (NIR) biological window is useful due to less absorption and scattering in biological tissues^19,20^, and small disturbance from autofluorescence^21^. The NIR biological window is separated into NIR-I (650–950 nm), NIR-II (1000–1350 nm), and NIR-III (1500–1800 nm). The NIR-II window is appropriate for *in vivo* deep imaging because of its lower absorption and scattering by biological tissues compared to NIR-I or NIR-III. The use of NIR-II expands the light penetration depth to ≈2–3 cm and allowed through-skull fluorescence imaging of the brain of mice^22^, whereas the penetration depth is limited to <1 mm in the UV and VIS wavelength ranges^23^. Recent works reported rare-earth-doped NaYF_4_ ceramics as NIR-II/III ratiometric nanothermometers^24^ for deep tissues^25^; however, this technique still required an observation depth-dependent calibration.

**Figure 1:**
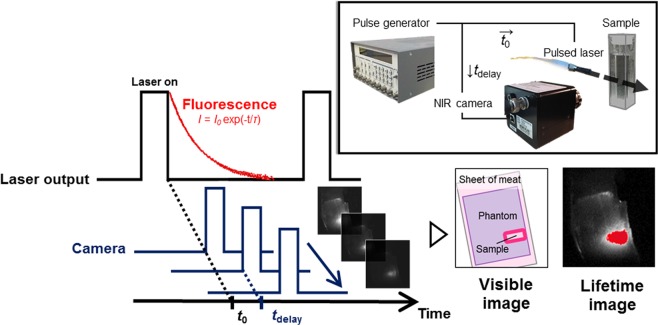
Schematic diagram of the experimental setup used for lifetime imaging. The pulsed laser and NIR camera are computer-controlled by a pulse generator to allow a time series of the fluorescence decay images for lifetime imaging. Analysis of the attenuation rate of fluorescence decay images obtained by the system allowed for measurement of the fluorescence lifetime at each pixel.

Here, we first attempt temperature imaging that is based on NIR-II fluorescence lifetime of rare-earth-doped NaYF_4_, which depends on the temperature of the surrounding media but not on observation depth. This material shows NIR-II emission with high efficiency^26^ and a relatively long lifetime^27,28^. To realize lifetime imaging of the NIR fluorophores, we constructed a time-gated imaging (TGI) system by controlling the delay of the timing between laser excitation and image acquisition^29^ (Fig. 1). Using an InGaAs camera that is sensitive to NIR-II^19^, the TGI system captures a pixel-level fluorescence decay curve that is simultaneously converted into fluorescence lifetime. Previous studies suggested that the TGI system also affords clear fluorescence images by minimizing autofluorescence of biological tissues in the NIR^28,30,31^. In addition, *in vivo* multiplexed imaging based on the fluorescence intensity and lifetime in NIR is available in the TGI system^32^. In this work, the capability of NIR-II fluorophore NaYF_4_ co-doped with Nd^3+^ and Yb^3+^ (NaYF_4_: Nd^3+^, Yb^3+^) as the lifetime-based thermometer was investigated. Temperature imaging of NaYF_4_: Nd^3+^, Yb^3+^ was demonstrated in a mimic of deep biological tissues to investigate the depth dependency of our TGI thermometry for NIR-II fluorescence.

## Results

### Fluorescence lifetime imaging of NaYF_4_: Nd^3+^, Yb^3+^ with changing temperature

**Figure 2:**
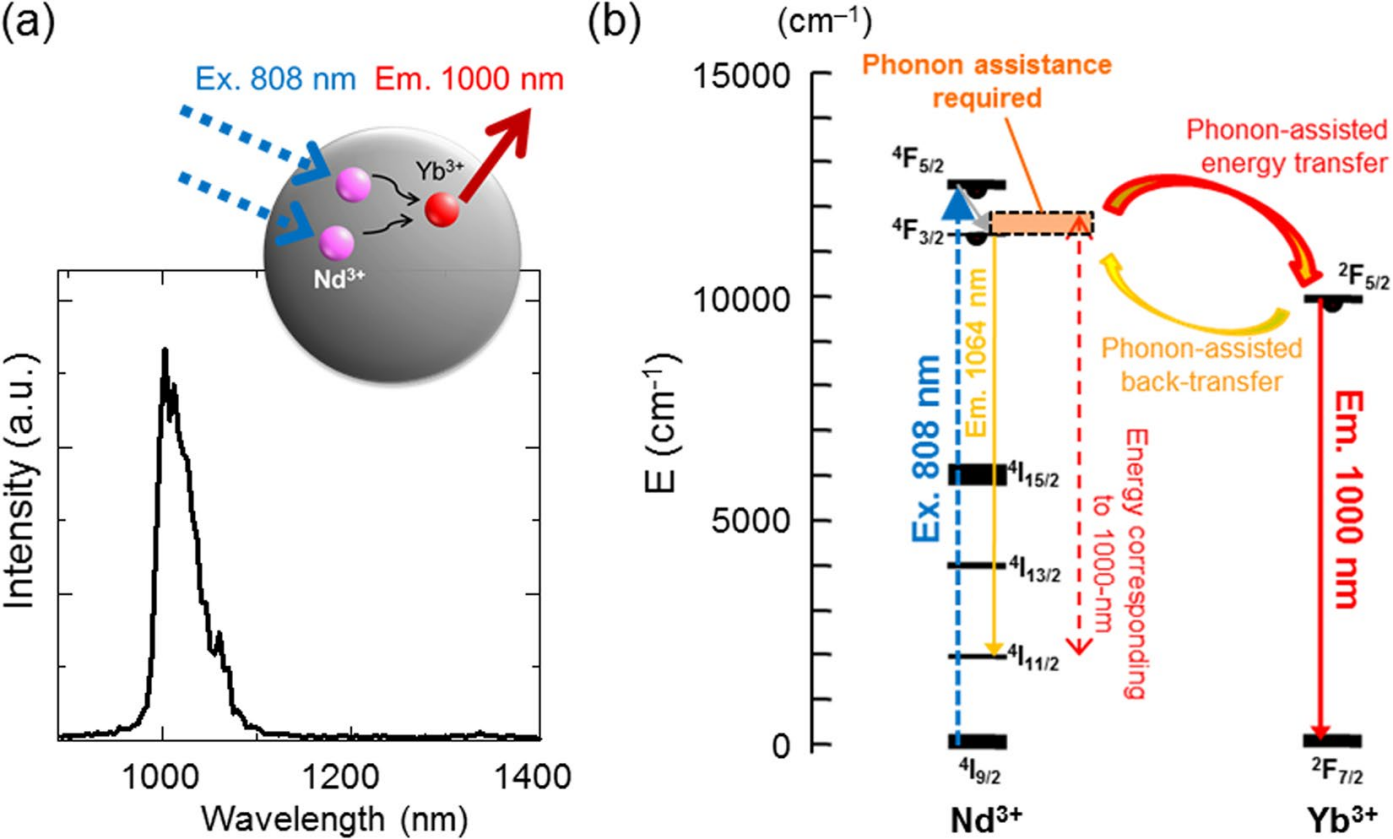
NIR fluorescence of NaYF_4_: Nd^3+^, Yb^3+^ sample under 808-nm laser excitation. (**a**) Emission spectrum of NIR fluorescence of Yb^3+^ (^4^F_5/2_ → ^4^F_7/2_) from NaYF_4_: Nd^3+^, Yb^3+^ at room temperature. (**b**) Schematic energy level diagram of NaYF_4_: Nd^3+^, Yb^3+^, which was used as the sensitizer excited by 808-nm laser and as the 1000-nm emitter.

First, elemental analysis of NaYF_4_: Nd^3+^, Yb^3+^ by energy-dispersive X-ray spectrometer (EDS) under field emission-type scanning electron microscope (FE-SEM) showed the material contained Y^3+^, Nd^3+^, and Yb^3+^ ions at a ratio of 60: 31: 9 (mol %) (Supplementary Fig. 1). The crystal phase of NaYF_4_: Nd^3+^, Yb^3+^ was mixture of α- and β-phases of NaYF_4_. Its NIR-II fluorescence was measured under excitation at 808 nm. Figure 2a shows the fluorescence spectra generated by the NaYF_4_: Nd^3+^, Yb^3+^ samples at room temperature. The spectrum shows a major emission band at approximately 1000 nm that is attributed to the Yb^3+^ (^2^F_5/2_ → ^2^F_7/2_) and a very small emission peak at 1064 nm derived from Nd^3+^ (Fig. 2b). The emission profile with the presence of the emission band generated by Yb^3+^ ions is clear evidence of an efficient energy transfer from Nd^3+^ to Yb^3+^ as described in detail previously^32^. The fluorescence lifetime of the sample was 460 μs at 25 °C as calculated from the fluorescence decay curve determined by infrared photomultiplier (Supplementary Fig. 2).

**Figure 3:**
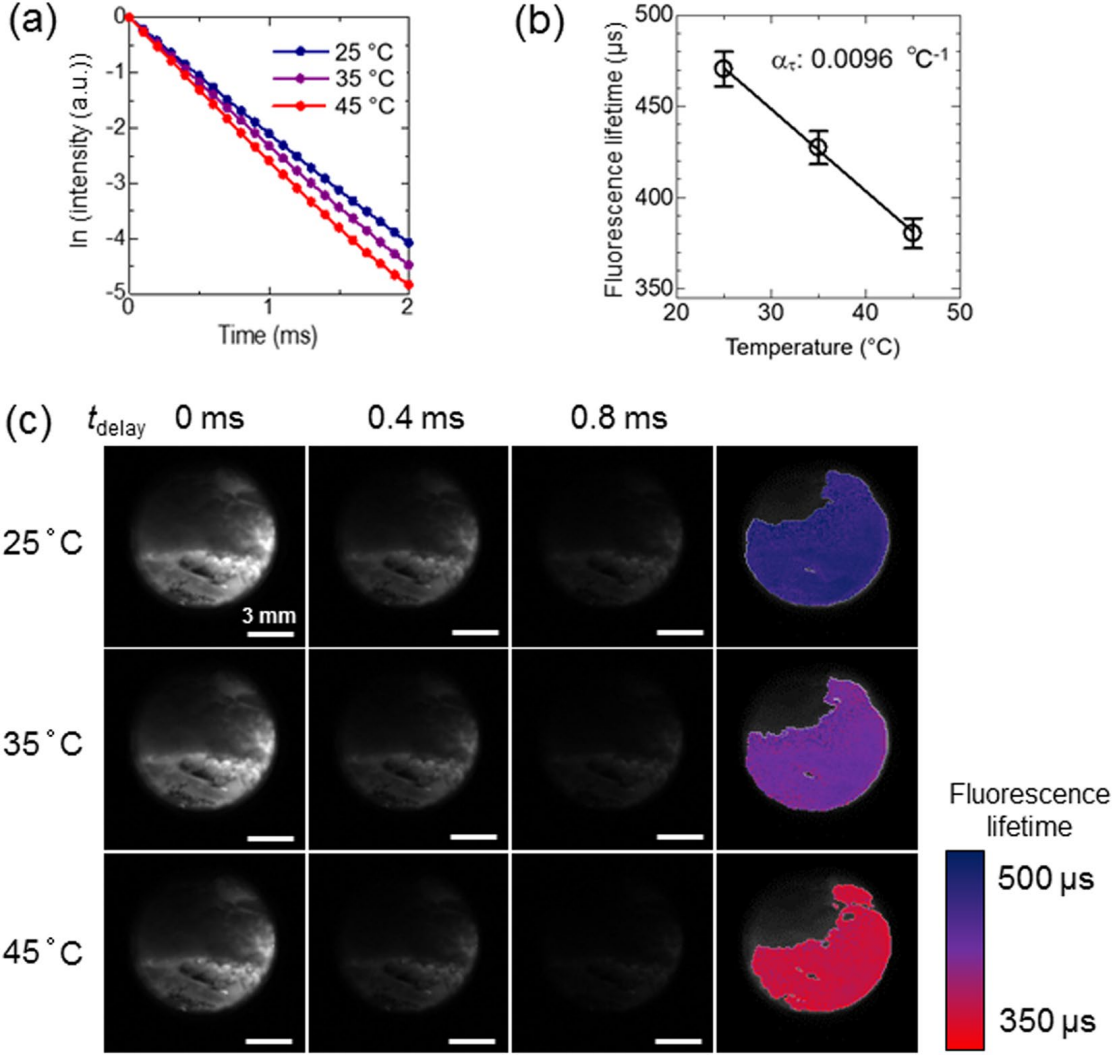
Thermal sensitivity of the fluorescence lifetime of NaYF_4_: Nd^3+^, Yb^3+^ detected by NIR camera. (**a**) Fluorescence decay curves of NaYF_4_: Nd^3+^, Yb^3+^ in optical cuvette at different experimental temperatures. (**b**) Calibration curve of temperature vs. fluorescence lifetime detected by NIR camera. (**c**) Examples of fluorescence images of NaYF_4_: Nd^3+^, Yb^3+^ at delay times of 0, 0.4, and 0.8 ms and calculated lifetime collected at each experimental temperature with excitation by 808-nm pulsed laser (5 W/cm^2^) from the right side.

Next, the fluorescence lifetime of the NaYF_4_: Nd^3+^, Yb^3+^ was detected on images acquired by the TGI system, where the temperature was controlled using a heater beneath the sample cuvette. According to the fluorescence decay curve of the NaYF_4_: Nd^3+^, Yb^3+^ (ex. 808 nm; em. 1000 nm) detected by NIR camera (Fig. 3a), its fluorescence lifetime was calculated as *τ* = $\int {I}_{em}({t}_{{\rm{delay}}}){\rm{d}}t/{I}_{0}$, where *I*_*em*_(*t*_delay_) is the emission intensity after the delay time (*t*_delay_) with respect to the excitation pulse, and *I*_0_ is the emitted intensity at *t*_delay_ = 0 s. The relationship between the calculated fluorescence lifetime and the grey values, which are the signal intensity levels at each pixel in greyscale images captured by the NIR camera, at a delay time of 0 ms was investigated on the biplots for each pixel to optimize the range of delay time used for calculation (Supplementary Fig. 3). The accuracy of fluorescence lifetime calculation at low grey values (15–20 at the delay time of 0 ms) was highest when the analysis was made by the data of delay times from 0 ms to 0.4 ms. Essentially, the plot contains two factors for analysis. One is the number of the delay-time points to be used for the analysis. If the points are more, that should give better analysis because of the averaging effects for avoiding the error due to the fractuation of the data. The other factor is the time range of the analysis. If the latter time points are contained, that causes more introduction of the errors due to worse signal-to-noise ratio at late timings. Accordingly, earlier time-range only contains less data to cause error and longer time range contains more noise to do so. Finally, medium range gives the best accuracy of the analysis as shown in the Supplementary Fig. 3b. Even in this case, the standard deviation of the calculated lifetime was over ±3.0% from the average at each temperature for pixels with grey values ≤15 (as an average of ten images for each condition) at the delay time of 0 ms (Supplementary Fig. 4a). These pixels with weak signals were excluded from the region of interest (ROI) of the temperature mapping. In addition, the biplots (Supplementary Fig. 3a) revealed that the lifetime was overestimated at the pixels with high grey values. The fluorescence lifetime of the pixels with grey values > 65 (*t*_delay_ = 0 ms) was overestimated (>3.0% over 460 μs, the lifetime determined by an infrared photomultiplier; Supplementary Fig. 4b) due to exceeding the number of photons over the linear dynamic range of the camera. This overestimation was solved by shifting the delay times for lifetime calculation from 0.1−0.5 ms to 0.4−0.8 ms (Supplementary Fig. 5). To obtain accurate data on the pixels with strong fluorescence intensities, the fluorescence lifetime of the pixels with grey values of >65 at *t*_delay_ = 0 ms was calculated from the values at *t*_delay_ of ≥0.1 ms at the same pixels. If the grey values at *t*_delay_ = 0.1 ms was >65, the fluorescence lifetime of the pixels was calculated from the value of longer delay times at the same pixel. Images of the fluorescence lifetime distribution of phosphor were successfully obtained by using the decay of NIR fluorescence at each pixel collected by the TGI system.

As shown in Fig. 3c, as the measurement temperature increased, the fluorescence lifetime of phosphor decreased markedly. The average fluorescence lifetime decreased from 470 ± 11 μs (25 °C) to 390 ± 12 μs (45 °C) (Fig. 3b). The calculated thermal coefficient, *α*_*τ*_, which represents the relative thermal sensitivity of the fluorescence lifetime, using the data obtained by the TGI system was 0.0096 °C^−1^. These results suggest that the use of NaYF_4_: Nd^3+^, Yb^3+^ is beneficial when applied to thermometry because its fluorescence lifetime is highly sensitive to temperature. Furthermore, the NIR camera in the TGI system detected the thermal dependence of the fluorescence lifetime. Thus, the fluorescence lifetime imaging detected by the TGI system in the NIR biological window could function as a thermometer.

### Temperature mapping by TGI

**Figure 4:**
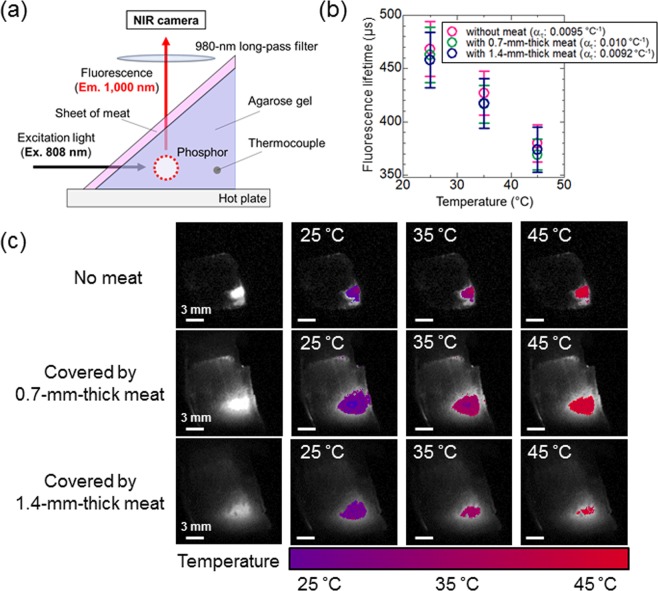
Thermal images obtained by calculation from the calibration curves of the thermally sensitive phosphor in Fig. 3. (**a**) Schematic representation of the biological deep imaging experiment using agarose gel and thin meat sheets. A hot plate was used as a heat source. (**b**) Thermal sensitivity of NaYF_4_: Nd^3+^, Yb^3+^ at each experimental condition. (**c**) Thermal images of NaYF_4_: Nd^3+^, Yb^3+^ while heating the biological tissue phantom from 25 °C to 45 °C with and without meat. Integration times: the sample without meat, 30 μs; the sample with one or two sheets of meat, 1000 μs. Laser power: 20 W/cm^2^.

Multiplexed NIR imaging was performed with a combination of NaYF_4_: Nd^3+^, Yb^3+^ and the TGI system. NaYF_4_: Nd^3+^, Yb^3+^ was inserted into agarose gel, which was used as a mimic of a biological phantom, on a hotplate to evaluate the temperature-dependent change in the fluorescence lifetime (Fig. 4a). NIR fluorescence images of NaYF_4_: Nd^3+^, Yb^3+^ in the phantom are shown in Fig. 4c. The thermal coefficient (*α*_*τ*_) in this experiment was calculated from the average values of fluorescence lifetime images at the ROI and was approximately 0.0093 °C^−1^, similar to the cuvette experiment (Supplementary Fig. 6). These results suggest that the fluorescence lifetime images calculated from the decay for each pixel can be converted to thermal images (Fig. 4c) by using the calibration curve between the temperature and fluorescence lifetime obtained by the TGI system (Supplementary Fig. 6). This indicates that the NaYF_4_: Nd^3+^, Yb^3+^ sample can be used as a lifetime-based thermometer in deep biological tissues because the emission of NaYF_4_: Nd^3+^, Yb^3+^ is not disturbed by water overtone absorbance.

Subsequently, 0.7-mm-thick sheets of meat were used to vary the depth of the phantom object. In this setting, with increased attenuation of excitation and emission lights, temperature imaging was carried out in a similar manner to the previous experiments by correcting the conversion from the lifetime to temperature based on the data shown in Supplementary Figs 4 and 5. The measured temperatures of the scattered parts and the ROI containing NaYF_4_: Nd^3+^, Yb^3+^ showed almost the same values, suggesting that the expanded area with fluorescence on the meat is caused by the scattering of fluorescence but not by excitation light. In the case of covering the phantom containing the NaYF_4_: Nd^3+^, Yb^3+^ sample with two sheets of meat, the fluorescence intensity decreased by the attenuation of excitation and emission light. However, the temperature-dependent change in the fluorescence lifetime was detected in the same manner as the phantom with no or less meat (Fig. 4b,c). For example, the lifetime 400 μs is converted to 40.6, 38.5, and 38.9 °C while the uncertainty, the estimated minimal resolvable temperature given by the product of 1/*α*_*τ*_, which is attributed to the intrinsic properties of the material, and the δ*τ*/*τ*, which is the relative uncertainty of the thermometric parameter given by the experimental instrumentation, is 3.11~6.15 °C in the present study. The thermal sensitivities (*α*_*τ*_), which were calculated from the temperature dependence of the fluorescence lifetimes for each condition as shown in Fig. 4b, were 0.0095 °C^−1^ (without meat), 0.010 °C^−1^ (with 0.7-mm-thick meat), and 0.0092 °C^−1^ (1.4-mm-thick meat). The thermal sensitivity of the phosphor used was 0.0096 °C^−1^ and was almost constant as the observation depth was varied from 0 to 1.4 mm by the sheets of meat. Thus, the results indicated that the temperature can be measured on the images independent of depth by fluorescence lifetime thermometry using an NIR fluorophore and the TGI system. The designed experimental system of biological deep temperature imaging as demonstrated here will open the pathway to *in vivo* temperature imaging independent of fluorescence intensity and tissue depth.

## Discussion

The lifetime imaging of NIR-II fluorescence is advantageous for temperature imaging of deep regions because of its independence of the observation depth. Our recent work has realized a conventional ratiometric method of temperature imaging for deep biological tissues (≈2-mm depth) using two bands of fluorescence wavelength (1150 and 1550 nm) of NaYF_4_ co-doped with Yb^3+^, Ho^3+^, and Er^3+^ phosphors^25^. However, it still required a calibration by the depth due to a slight shift of absorption spectrum of water, which is contained in the tissues, by temperature change. The possible change in the optical loss by biological tissues should be taken care in *in vivo* deep temperature imaging such as the brain in mice^34^. In contrast, the lifetime-based method principally normalizes the original data of fluorescence intensity by the simultaneously obtained data with different delay time after cutting off the excitation irradiation. Therefore, this method shown in the present study does not need further calibrations by excitation power density or the observation depth for absolute temperature measurement. The most advantage of using rare-earth-based materials is their long lifetime at several microseconds. The long lifetime of the phosphors makes their lifetime measurable by the TGI system with conventional cameras for NIR-II. Basically, this fluorescence lifetime sensing method is done by the measurement of luminescence lifetimes of the excited energy levels over the temperature range of investigation as previously mentioned^12^. The fluorescence lifetime of NaYF_4_: Nd^3+^, Yb^3+^ increases if the Nd^3+^ → Yb^3+^ energy transfer was enhanced, while it decreases when the Nd^3+^ ← Yb^3+^ energy back-transfer process was activated^32^. The temperature-dependent change in the fluorescence lifetime is possibly due to the dependence of phonon-assisted energy transfers between Nd^3+^ and Yb^3+^ (Fig. 2b) on temperature. In fact, the mechanism is known to be a pure and simple one. It is always a mixture of resonant and phonon-assisted energy transfer, multi-phonon relaxation and back transfers. For wider range of temperature for discussing the characteristics of the thermometry, various kinds of analysis will be required. Although the inaccuracy of 3.0% was accepted for the lifetime calculation to make the temperature images in this article, it is improved to less than 1.5% if the pixels with low grey values would be excluded from the ROI (Supplementary Fig. 4a). Previous studies have shown that visible emission by upconversion of rare-earth-based nanoparticles is available for sub-tissue thermal sensing (1-mm depth) by lifetime analysis^35^. Ratiometric fluorescence thermometry was also reported using NIR emission bands in shorter NIR wavelength (762 and 825 nm) of a material co-doped with Nd^3+^ and Yb^3+^ ions^36^. Our methods that use the lifetime of NIR emission at longer wavelength have an advantage to do thermal sensing for deeper regions at several millimetres depth. Because we used micro-sized particle that has small surface area in the present study, its fluorescence properties including the lifetime were not affected by the environment, such as pH, surrounding the surface of the particle.

Note that a complete linearity between the number of photons and detected grey values is required for determining fluorescence intensity from the decay images. The linearity is possibly distorted by temporal shortage of electrons in the sensor to cause a negative bias. The electrons are concentrated in the negative bias when very strong pulse comes to decrease the output of modulated phenomena. If not suffered by the strong pulse, the camera output is linear for luminescence observation. Only with this great excitation earlier timing signals are suppressed. The lifetime-based temperature imaging is available using the grey value data of appropriate delay times in the linear range for each pixel. The appropriate delay times should be found for each pixel using the biplots to investigate the relationship between detected fluorescence intensities and calculated fluorescence lifetime in the TGI system. The system reported here is potentially able to show the distribution of temperature in objects. The observation depth of our new temperature imaging technique is several millimeters that may not be perfect for the clinical use. However, this new method based on the long fluorescence lifetime of the NIR-II fluorophores is potentially available as a contactless biomedical imaging tool for understanding the mechanisms of temperature distribution changes in animal models. The error of temperature estimation is still large (3~4 °C) for the samples covered by meat. The cause of the large error is not likely to be the power of excitation because the error was not dependent of the thickness, but is possibly the difference in the integration time to collect images of the fluorescence decay, even though the thermal sensitivity of the fluorescence lifetime is independent of meat thicknesses. The thermal sensitivity of our fluorescence probe was calculated as the thermal coefficient at 0.0096 °C^−1^, which is in a similar range to the previously reported high-sensitive thermometers based on the fluorescence lifetime^35,37^. The probes with high thermal sensitivity, along with high quantum yield and higher biocompatibility, will realize definitive optical and contactless sensing of the temperature in biological organs and help us to understand the mechanism of biological phenomena controlled by temperature distributions.

Some limitations of our study merit discussion. First, we used micro-sized particle composed of a mixture of α- and β-phase NaYF_4_. With the method for synthesising the particles, it is very difficult to control the single phase formation. Since the singularity of the phase is not a focus of our study, we gave up this control to seek for. We suppose it does not affect onto the story of this manuscript. Further investigation is required to determine how the temperature-dependent fluorescence decay can be observed on a single phase NaYF_4_: Nd^3+^, Yb^3+^ nanoparticle, which is suitable for biomedical thermometry application, by NIR cameras with the TGI system. Second, we used a long-pass filter with a cut-on wavelength at 980 nm. We chose this filter to cut the photo-excitation pulse as well as possible; however, it also cut a part of Yb^3+^ emission spectrum. Ideally, if one has a sharper cutting filter with better optical density, that results in more convenient. The effect of the excitation pulse might be enhanced when nanoparticles would be used as a thermometer because of their lower emission quantum yield than micro-sized powders. Further investigation with optimized optical system with more appropriate spectral filters is also needed. Finally, the uncertainty of the estimation of temperature reported here (3.11~6.15 °C) is not good compared to previously reported nanothermometers^25,33^. Further experiments are currently underway for optimising dopant concentration and core/shell architecture^31^ to enhance the thermal sensitivity of the NIR fluorescence lifetime and to improve the accuracy of the temperature measurement.

## Conclusions

In summary, we successfully visualized temperature changes by using the TGI system for analysing the decay time of NIR-II fluorescence of NaYF_4_: Nd^3+^, Yb^3+^ particles in biological deep tissue under 808-nm laser excitation. The results obtained at different thermometer depths, controlled by covering the agarose gel with sheets of meat, showed that thermometry regardless of depth could be realized. The thermal sensitivity of the phosphor used for the measurement was 0.0096 °C^−1^, and the sensitivity of its fluorescence lifetime was almost constant (−0.0092 ~ −0.010 °C^−1^) while estimating the fluorescence decay through varying meat thicknesses from 0 to 1.4 mm. Therefore, a thermal imaging system comprised of a rare-earth-doped fluorophore and the TGI system can image deep tissue temperatures without the issues introduced by tissue in the optical pathway or laser power. Future work will focus on the development of novel NIR-II fluorophores with high quantum yields, high thermal sensitivities, and high biocompatibilities, all of which could lead to further advances towards *in vivo* optical temperature imaging.

## Methods

### Materials

Yttrium(III) nitrate hexahydrate (Y(NO_3_)_3_·6H_2_O) and sodium fluoride (NaF) were purchased from Kanto Chemicals (Tokyo, Japan). Neodymium(III) nitrate hexahydrate (Nd(NO_3_)_3_·6H_2_O) and ytterbium(III) nitrate (Yb(NO_3_)_3_·6H_2_O) were purchased from Sigma-Aldrich (St. Louis, MO, USA). Agarose powder was purchased from Nacalai Tesque Inc. (Kyoto, Japan). All the reagents were used without further purification.

### Synthesis and optical characterization of rare-earth-doped NaYF_4_

NaYF_4_: Nd^3+^, Yb^3+^ was synthesized by a standard co-precipitation method^38^. Y(NO)_3_·6H_2_O (6.0 mmol), Nd(NO)_3_·6H_2_O (3.0 mmol), and Yb(NO)_3_·6H_2_O (1.0 mmol) were dissolved in 10 mL of distilled water. The mixture solution was dropped into 40 mL of an aqueous solution of NaF (60 mmol) and stirred for 1 h at 75 °C. After stirring, the precipitate was collected by centrifugation (20,000 *g*, 10 min, ×3). The sample was then dried at 80 °C for 24 h. Subsequently, the samples were treated with NH_4_F (800 mg) for 1 h at 550 °C to yield NaYF_4_ co-doped with 30 mol% Nd^3+^ and 10 mol% Yb^3+^. The crystalline phase of synthesized NaYF_4_: Nd^3+^, Yb^3+^ was analysed by using RINT-TTR III (Rigaku, Japan). Elemental component of the NaYF_4_: Nd^3+^, Yb^3+^ was analysed by FE-SEM/EDS (S-4200; Hitachi High-Technologies Co., Tokyo, Japan). Fluorescence spectra of the NaYF_4_: Nd^3+^, Yb^3+^ under 808-nm excitation were detected with a spectrometer (NIRQuest; Ocean Optics Inc., Dunedin, FL, USA). Fluorescence lifetime of the sample at room temperature (25 °C) was investigated by using an optical parametric oscillator (Surelite II-10; Continuum Inc., San Jose, CA, USA) pumped by a frequency tripled Nd-doped yttrium aluminum garnet laser operating at 355 nm. The optical parametric oscillator provides 5 ns pulses at 808 nm wavelength with an average energy of 9.5 mJ and a repetition rate of 10 Hz. Time evolution was detected by an infrared photomultiplier (H10330C; Hamamatsu Photonics Co. Ltd., Shizuoka, Japan) connected to a digital oscilloscope (TDS2024C; Tektronix Inc., Beaverton, OR, USA) with a 980-nm long-pass filter (#86–248; Edmund Optics Inc., Barrington, NJ, USA) to detect only fluorescence.

### Time-gated imaging of rare-earth-doped ceramics nanoparticle

The TGI system, composed of a pulsed laser, an NIR camera, and a pulse generator, was used to acquire fluorescence decay and lifetime images, as schematically shown in Fig. 1. A custom-built pulsed laser diode (wavelength: 808 nm; power: 4 W) was used to generate 10 ms pulses at a repetition rate of 20 Hz. The pulse-to-pulse separation was set to 40 ms, during which the fluorescence of the phosphors disappears completely. A time series of fluorescence decay images was obtained with an NIR camera (ARTCAM-0016TNIR; Artlay Co. Ltd., Tokyo, Japan). A 980-nm long-pass filter was placed in front of the NIR camera. A digital delay/pulse generator (DG535; SRS Inc., Sunnyvale, CA, USA) connected the laser and the camera to trigger them at a delayed time (*t*_delay_). To obtain the fluorescence decay curves, a series of fluorescence images (8-bit) was acquired, where *t*_delay_ ranged from 0 to 1.0 ms in increments of 0.1 ms. For lifetime measurements, NaYF_4_: Nd^3+^, Yb^3+^ (30 v/v% in water) in an optical glass cuvette (PSK-10; Sansyo Co. Ltd., Tokyo, Japan) was placed in a temperature-controlled cuvette holder (qpod-2e, Ocean Optics Inc., Dunedin, FL, USA) and imaged in the TGI system, and the temperature was varied from 25 °C to 45 °C. For temperature imaging, a phantom consisting of NaYF_4_: Nd^3+^, Yb^3+^-embedded agarose gel (3%) was placed on a hot plate (CH-180; As One Co. Ltd., Osaka, Japan). A thermocouple (DS-2000-0121; As One Co. Ltd., Tokyo, Japan) was inserted into the phantom to measure the temperature at the position of the embedded fluorophores. Note that the vertical position of the thermocouple and the fluorophores was the same. After heating the phantom with the hot plate to 45 °C, the fluorescence decay of the phosphors was recorded using the TGI system during the thermal relaxation. To study the influence of observation depth in biological tissues, one or two sheets of commercial raw pork meat (0.7 mm thick each) were placed over the phantom, and the temperature imaging was performed through the sheets. The influence of depth on temperature distribution measurements was evaluated by calculating the normalized lifetime thermal coefficient, which was defined as ${\alpha }_{\tau }=|d{\tau }^{{\rm{nor}}}{(T)}_{\tau }/dT|$, where ${\tau }_{f}^{nor}(T)$ is the fluorescence decay time at temperature *T* normalized to the room temperature value (i.e. ${\tau }_{f}^{{\rm{nor}}}(T)={\tau }_{f}(T)/{\tau }_{f}({\rm{25}}\,^\circ {\rm{C}})$)^37^.

## Supplementary information

Supplementary Figures 1-6

## Data Availability

The data required to reproduce these findings are available to download from https://data.mendeley.com/datasets/8ysrmm83hb/draft?a=e86a71d8-1749-40be-b3ff-a790e97b6a66.
