# Supplementary material for: Biological Deep Temperature Imaging with Fluorescence Lifetime of Rare-Earth-Doped Ceramics Particles in the Second NIR Biological Window
Source: Sci Rep. 2019 Sep 5;9:12806. doi: 10.1038/s41598-019-49291-x (PMC6728332; doi:10.1038/s41598-019-49291-x)
Supplement: Supplementary file 1 — Supplementary Figures 1-6 [file 41598_2019_49291_MOESM1_ESM.docx]

**Supplementary Information**

**Biological Deep Temperature Imaging with Fluorescence Lifetime of Rare-Earth-Doped Ceramics Particles in the Second NIR Biological Window**

Takumi Chihara^1^, Masakazu Umezawa^1^*, Keiji Miyata^1^, Shota Sekiyama^1^, Naoki Hosokawa^1^, Kyohei Okubo^1^, Masao Kamimura^1,2^ and Kohei Soga^1,2^*

^1^Department of Materials Science and Technology, Faculty of Industrial Science and Technology, Tokyo University of Science, 6-3-1 Niijuku, Katsushika, Tokyo, Japan

^2^Imaging Frontier Center (IFC), Research Institute for Science and Technology (RIST), Tokyo University of Science, 2641 Yamazaki, Noda, Japan

*Corresponding Authors


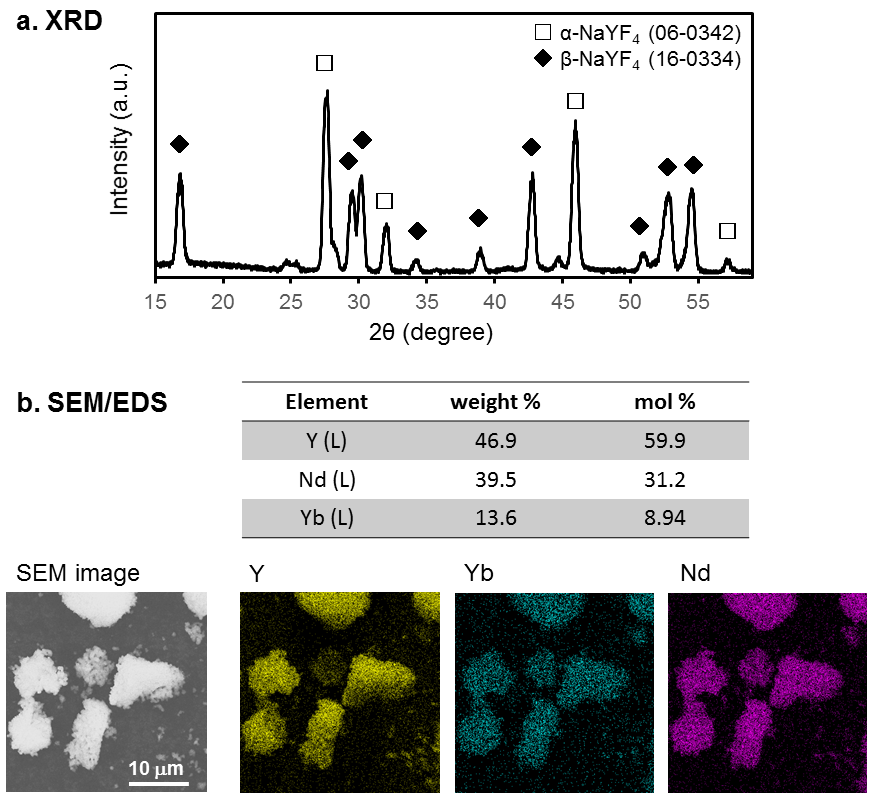


**Supplementary Figure 1 |** **Characterisation of NaYF_4_:Nd^3+^,Yb^3+^ used in this study. a,** The pattern of X-ray diffraction (XRD). The data shows that the material was the mixture of α- and β-NaYF_4_. **b,** The ratio of Nd^3+^ and Yb^3+^ concentrations in NaYF_4_ determined by field emission-type scanning electron microscopy/energy dispersive X-ray spectroscopy (SEM/EDS). Averaged EDS spectrum was obtained from an entire area in the images. ZAF correction method was used to quantify mol (atomic) % of elements in the particles.


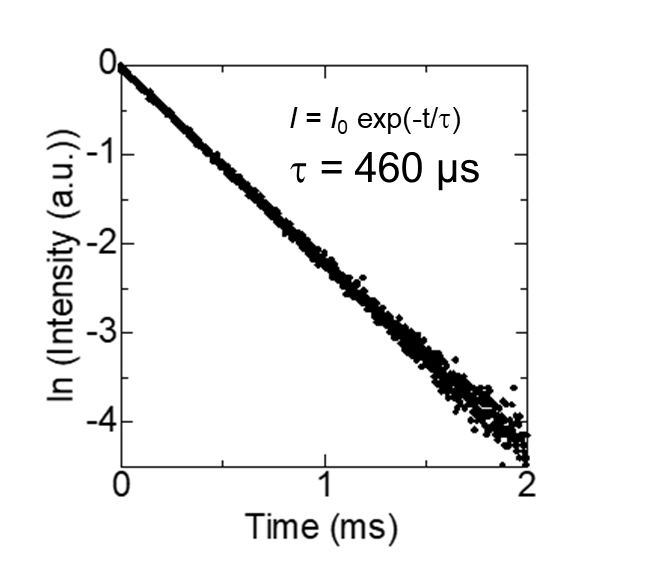


**Supplementary Figure 2 |** **Fluorescence decay curve of NaYF_4_:Nd^3+^,Yb^3+^ in optical cuvette determined by infrared photomultiplier at 25** °C**.** Calculated fluorescence lifetime of sample was 460 μs.


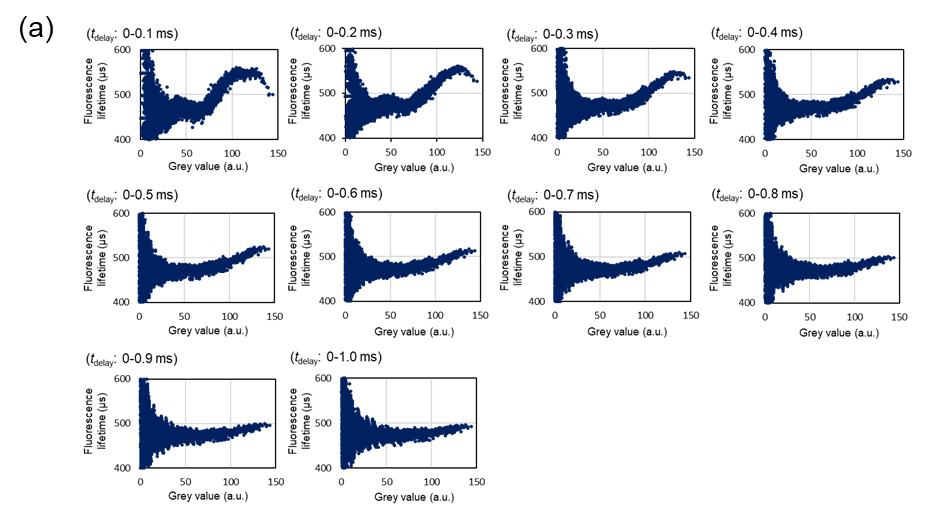


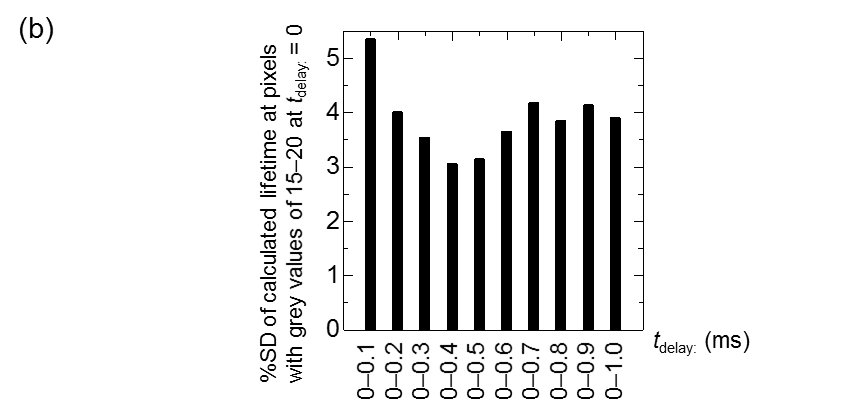


**Supplementary Figure 3 |** **The relationship between the calculated fluorescence lifetime and the grey values (fluorescence intensity) at *t*_delay_ = 0 ms. a,** Biplots of the grey values (fluorescence intensity) and calculated fluorescence lifetime from the different ranges of delay time (*t*_delay_) for each pixel. **b,** The percentile standard deviation (%SD) of the calculated lifetime to the average for the pixels with grey values of 15−20 (*t*_delay_ = 0 ms).


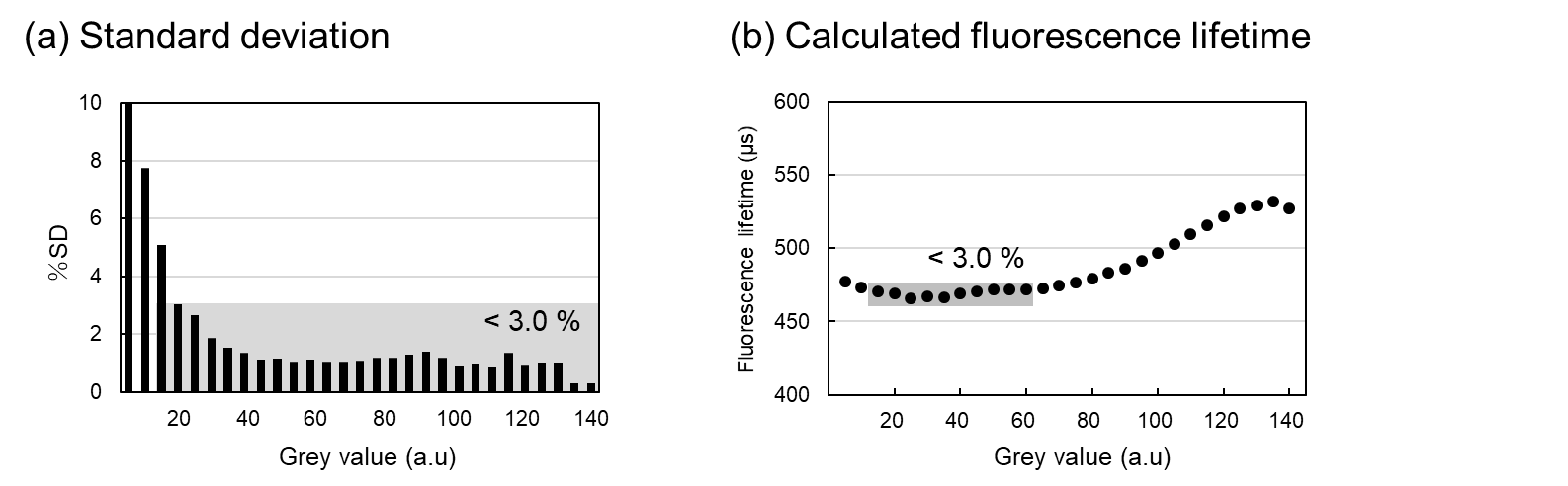


**Supplementary Figure 4 | The relationship between the errors of fluorescence lifetime calculated by the data (*t*_delay_ = 0–0.4 ms) and the grey values (fluorescence intensity) at *t*_delay_ = 0 ms at 25 °C.** **a,** The percentile standard deviation (%SD) of calculated lifetime to the average within each grey value range. **b**, Calculated average fluorescence lifetime at each grey value range. The fluorescence lifetime detected by infrared photomultiplier (*τ* = 460 μs) was used as the reference, of which the ± 3.0% was marked.


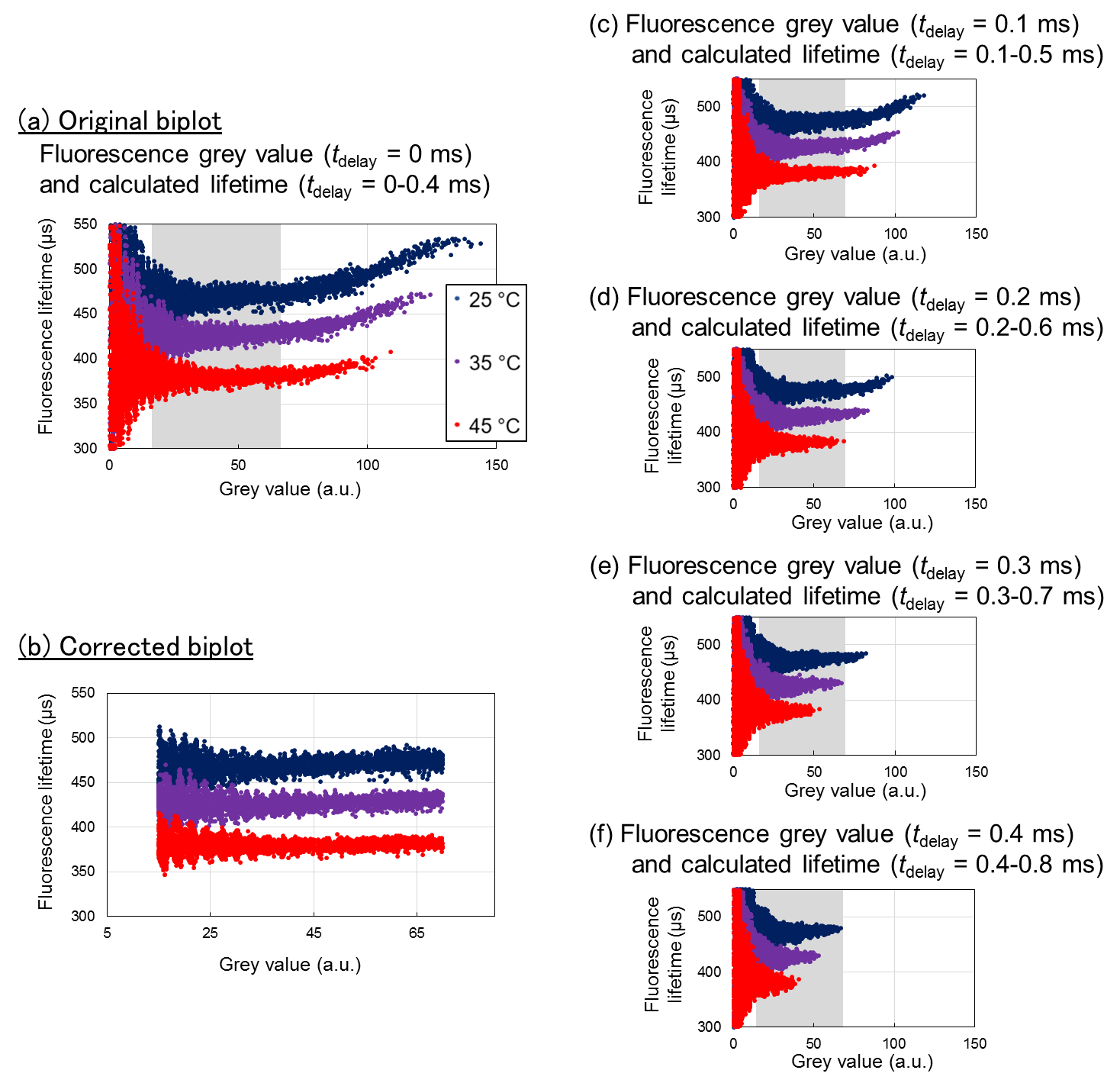


**Supplementary Figure 5 | Biplots of calculated fluorescence lifetime and grey values (fluorescence intensity). a,** Original biplot showing the relation between the grey values at the delay time (*t*_delay_) of 0 ms and the lifetimes calculated from the data within delay time of 0−0.4 ms. **b**, Corrected biplot used to obtain the lifetime-based images shown in Fig. 3c, which is prepared by original biplots (a, c−f). **c−f,** Biplots showing the relation between the grey values at the different delay times at (c) 0.1 ms, (d) 0.2 ms, (e) 0.3 ms, and (f) 0.4 ms and the lifetimes calculated from the delay time data at (c) 0.1–0.5 ms, (d) 0.2–0.6 ms, (e) 0.3–0.7 ms and (f) 0.4–0.8 ms for each pixel to prepare the corrected biplot (b).


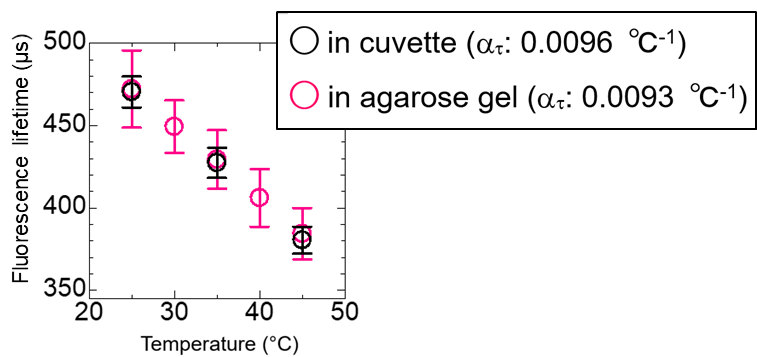


**Supplementary Figure 6 | Temperature-dependent change in fluorescence lifetime of NaYF_4_:Nd^3+^,Yb^3+^ in an optical cuvette and agarose gel.** While the fluorescence lifetime of the phosphor depends on the temperature, the rate of temperature-dependent change in the fluorescence lifetime is constant regardless of other factors.
